# Identification of Diversity-Generating Retroelements in Human Microbiomes

**DOI:** 10.3390/ijms150814234

**Published:** 2014-08-15

**Authors:** Yuzhen Ye

**Keywords:** DGR, human microbiome, DGRscan, reverse transcriptase (RTase), template region (TR), variable region (VR)

## Abstract

Diversity-generating retroelements (DGRs) are a unique family of retroelements that confer selective advantages to their hosts by accelerating the evolution of target genes through a specialized, error-prone, reverse transcription process. First identified in a *Bordetella* phage (BPP-1), which mediates the phage tropism specificity by generating variability in an involved gene, DGRs were predicted to be present in a larger collection of viral and bacterial species. A minimal DGR system is comprised of a reverse transcriptase (RTase) gene, a template sequence (TR) and a variable region (VR) within a target gene. We developed a computational tool, DGRscan, to allow either *de novo* identification (based on the prediction of potential template-variable region pairs) or similarity-based searches of DGR systems using known template sequences as the reference. The application of DGRscan to the human microbiome project (HMP) datasets resulted in the identification of 271 non-redundant DGR systems, doubling the size of the collection of known DGR systems. We further identified a large number of putative target genes (651, which share no more than 90% sequence identity at the amino acid level) that are potentially under diversification by the DGR systems. Our study provides the first survey of the DGR systems in the human microbiome, showing that the DGR systems are frequently found in human-associated bacterial communities, although they are of low incidence in individual genomes. Our study also provides functional clues for a large number of genes (reverse transcriptases and target genes) that were previously annotated as proteins of unknown functions or nonspecific functions.

## 1. Introduction

The diversity-generating retroelement (DGR) was first identified in a *Bordetella* phage (BPP-1), which was shown to mediate the phage tropism specificity by generating variability in a gene involved (the target gene, *mtd*), resulting in massive sequence variation in the phage’s receptor-binding protein, Mtd, encoded by the target gene [1]. Central to this diversity-generating process (known as retrohoming) is a reverse transcriptase-mediated exchange between two repeats: one serves as a donor template (TR) and the other as a recipient of variable sequence information (VR). During this process, adenines encoded in the TR are replaced randomly in the VR (so, the nucleotide substitution is A-specific). Accordingly, a minimal DGR system that is functional should consist of a TR, a VR (the VR is homologous to the TR and forms part of the coding sequence of the variable protein, e.g., Mtd) and a gene that encodes for a reverse transcriptase (RTase or RT). Structural features important for target-site recognition have been revealed [2]. A gene called *avd* that encodes accessory variability determinant (Avd) protein is often found with other core DGR elements. Recently, the tertiary structure of Avd was solved, and mutational analysis revealed a strict correspondence between retrohoming and interaction of Avd with RT, suggesting that the RT–Avd complex is important for DGR retrohoming [3].

DGRs were predicted to be present in a larger collection of viral and bacterial species [4]. However, in a recent systematic study, only 155 DGR elements were identified in more than 6000 prokaryotic and phage genomes, indicating a surprisingly low incidence of DGR systems in sequenced genomes [5]. It was found that some genomes carrying the DGR systems may have multiple target genes that are associated with the DGR systems, with target genes far apart in the genomes, but all have a core system consisting of a TR, a VR and a RTase gene [5]. DGR systems remain largely mysterious: The detailed mechanism is largely unknown, and many of the target genes diversified by the DGR systems have no known functions, although for a significant proportion of these genes, their protein products can be predicted to have the *C*-lectin fold [6]. The biological functions of most of the predicted DGRs remain to be determined, but it seems likely that they play similar roles as the DGR found in the *Bordetella* phage, so providing a mechanism for adapting to an unpredictable host, environment or both.

DGR systems are the only known source of massive protein sequence variation in prokaryotes. They were identified as one of the two sources for the extreme interpersonal diversity of human gut viruses [7] (the other source is associated with the CRISPR arrays [8]). Arambula *et al*. [9] characterized a DGR in *Legionella pneumophila* within a horizontally acquired genomic island and identified related DGRs in *L. pneumophila* clinical isolates that encode unique target proteins with homologous VRs, demonstrating the adaptability of DGR components.

**Figure 1:**
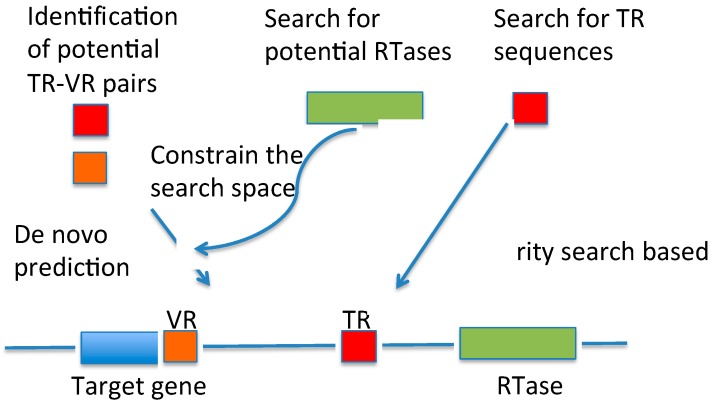
DGRscan provides both *de novo* and homology-based predictions of DGR systems. DGRscan scans a genomic sequence for potential TR–VR pairs, repeats that contain substitutions mostly involving adenines in the TR region. Predictions of potential RTase genes can also be utilized to constrain the search of TR–VR pairs, so that a *de novo* search is applied only in the neighborhood of predicted RTase genes for speedup. If TR sequences are given, DGRscan can be guided to look for DGR systems that have homologous TRs (and VRs) similar to the reference TRs.

Despite the striking ability of the DGR systems in introducing vast amounts of targeted diversity into protein-encoding DNA sequences via mutagenic homing and their potential biological impacts, the systematic study of the DGR systems showed a low incidence of this system in sequenced genomes [5]. Metagenomic projects have resulted in the accumulation of large amounts of genomic sequences, most from uncultured microbial organisms, providing us great opportunities for studying the DGR systems in diverse collections of microbial communities, in their natural environment. These projects have enabled us to ask questions, such as “Are DGR systems of also low incidence to be found in microbial communities?” A study of human-associated bacterial phages has resulted in the identification of ~30 DGR systems, each with a core system of RT, template and variable sequences. However, many more metagenomic sequencing datasets, including the human microbiome project (HMP) datasets [10,11], have not been explored for the diversity and the distribution of the DGR systems. To facilitate DGR discovery in metagenomes, we developed DGRscan, a computational tool for the identification of DGR systems (see Figure 1), and applied it to study the DGR systems in human microbiomes. Compared to DiGReF [5], the only existing software for DGR identification, DGRscan provides more options for the identification of DGR systems. In addition, DGRscan can be used for identifying incomplete DGR systems in fragmented contigs assembled from metagenomic sequences or even short metagenomic sequences using known template sequences as the reference, a feature that is important for its application to metagenomic datasets. The application of DGRscan to the HMP datasets resulted in the identification of many new DGR systems and putative target genes that are potentially under diversification of the DGR systems.

## 2. Results and Discussion

We first tested DGRscan on known DGR systems and then applied it to predict the DGR systems in the HMP datasets. For the DGR systems identified in the HMP datasets, we focused our analyses on the identified reverse transcriptases and target genes.

### 2.1. Testing of DGRscan on Known DGR Systems

We tested DGRscan on a human virome dataset [7]. We used 155 RTases identified in [12] as the reference to identify potential RTs in 29 human virome contigs, which were used to constrain the *de novo* prediction of DGR systems in these contigs. As shown in Table 1, except for the contig (gi|377806399|gb|JQ680376.1|) that has a TR fragment of only 44 bps, DGRscan was able to predict TRs in the remaining 28 contigs that were reported to contain DGR systems (DGRscan only reports TRs that are of at least 60 bps; see the Experimental Section). All TRs predicted by DGRscan overlapped with the previously reported TRs, although they may vary in the exact boundaries. We note that for a few cases, TRs reported in the previous study were truncated. However, DGRscan was able to recover the longer TRs (e.g., in contigs gi|377806018|gb|JQ680360.1| and gi|377806292|gb|JQ680371.1|, highlighted in bold in Table 1).

**Table 1:** Identification of template regions (TRs) by DGRscan for the human virome dataset.

Sequence (gi)	Previous Study [7]		DGRscan	
	Length (bp)	Start–End	Length (bp)	Start–End
gi|377805826|gb|JQ680351.1|	101	22,583–22,683	112	22,869–22,980
gi|377805855|gb|JQ680352.1|	139	7998–8136	131	7998–8128
gi|377805862|gb|JQ680353.1|	89	14,041–14,129	128	14,041–14,168
gi|377805758|gb|JQ680349.1|	97	39,459–39,555	126	39,449–39,574
gi|377805877|gb|JQ680354.1|	128	46,739–46,866	128	46,739–46,866
gi|377805785|gb|JQ680350.1|	101	17,450–17,550	128	17,450–17,577
gi|377805936|gb|JQ680355.1|	101	3782–3882	94	3782–3875
gi|377806015|gb|JQ680359.1|	116	4435–4550	126	4435–4560
gi|377805967|gb|JQ680357.1|	101	3148–3248	158	3091–3248
gi|377806003|gb|JQ680358.1|	128	5598–5725	128	5598–5725
gi|377805941|gb|JQ680356.1|	128	38,849–38,976	128	38,849–38,976
**gi|377806018|gb|JQ680360.1|**	**23**	**6556–6578**	**116**	**6463–6578**
gi|377806060|gb|JQ680361.1|	78	21,703–21,780	116	21,692–21,807
gi|377806090|gb|JQ680362.1|	121	4920–5040	122	4918–5039
gi|377806097|gb|JQ680363.1|	101	4306–4406	110	4306–4415
gi|377806107|gb|JQ680364.1|	121	11,625–11,745	122	11,620–11,741
gi|377806133|gb|JQ680365.1|	101	32,701–32,801	94	32,685–32,778
gi|377806170|gb|JQ680366.1|	101	8872–8972	113	8818–8930
gi|377806186|gb|JQ680367.1|	120	41,651–41,770	123	42,437–42,559
gi|377806226|gb|JQ680368.1|	121	27,017–27,137	126	27,012–27,137
gi|377806251|gb|JQ680369.1|	85	3464–3548	115	3464–3578
gi|377806260|gb|JQ680370.1|	115	25,676–25,790	115	25,676–25,790
gi|377806297|gb|JQ680372.1|	151	2108–2258	121	2108–2228
**gi|377806292|gb|JQ680371.1|**	**59**	**4471–4529**	**110**	**4471–4580**
gi|377806399|gb|JQ680376.1|	44	3993–4036	-	-
gi|377806374|gb|JQ680375.1|	101	23,576–23,676	75	23,569–23,643
gi|377806345|gb|JQ680374.1|	101	4412–4512	95	4420–4514
gi|377806301|gb|JQ680373.1|	101	20,259–20,359	133	20,252–20,384
gi|377806422|gb|JQ680377.1|	121	13,118–13,238	105	13,134–13,238

### 2.2. Identification of the DGR Systems Associated with the Human Microbiome

Application of DGRscan to the HMP datasets (*de novo* prediction using RTs as constraints) revealed a total of 765 DGR systems in the human microbiomes. The number of identified DGR systems is reduced to 271 after removing the redundancy using CD-HIT with a cutoff of 90% sequence identity [13] (DGR systems were clustered according to the RT genes they contain). We are able to double the number of DGR systems by using the human microbiome data: there were 155 DGR systems identified from the reference genomes [12] (the number is reduced to 129 when using 90% sequence identity to cluster the RT sequences) and 29 DGR systems (24 at 90% non-redundant) identified from the human virome data [7].

We note that about half of these genes (130 out of 271; 48%) were shown to be “hypothetical proteins” in the HMP annotations. For the remaining genes, the HMP annotation shows that 64 genes encode “RNA-directed DNA polymerase”, 22 encode “reverse transcriptase family protein”, 18 encode “retron-type reverse transcriptase”, 11 encode “hypothetical protein bfra3_11896” and six encode “hypothetical protein bfra3_13530”. However, knowing that a gene encodes for a “RNA-directed DNA polymerase” or “a reverse transcriptase” is not really that helpful, because reverse transcriptases are involved in many different biological processes (e.g., replication of retroviruses and retrotransposition in Eukaryotes) [14], not to mention that the PFAM domain RVT_1 for reverse transcriptase contains a huge number of sequences (>170 K) [15]. Using the genomic context information, we were able to assign functions and, more importantly, the functional context for these 271 reverse transcriptases; *i.e.*, they are reverse transcriptases involved in the DGR systems.

**Figure 2:**
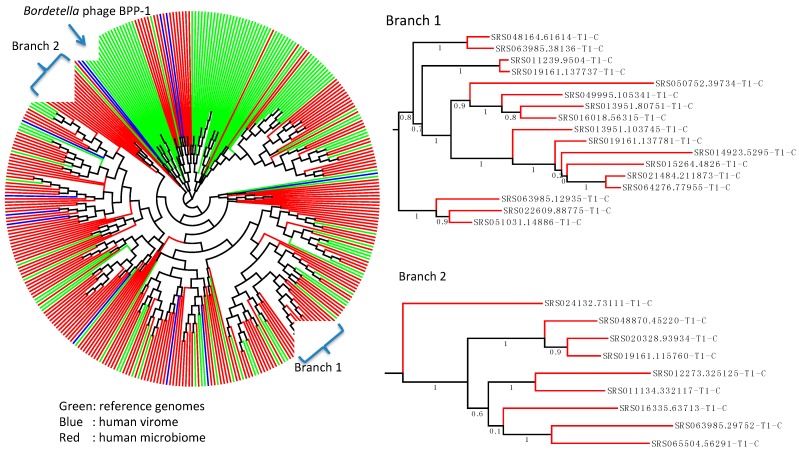
A phylogenetic tree of the reverse transcriptases identified from the reference genomes (shown in green) [12], human virome (blue) [16] and human microbiomes (this study; red). The RT identified from *Bordetella* phage BBP-1 is highlighted in the tree. The multiple alignment was done using MUSCLE [17]. The FastTree program [18] with default parameters was used to reconstruct the neighbor-joining tree, and the tree was visualized using the Archaeopteryx tree viewer [19].

Combing the RTs we identified from the HMP datasets and those identified from the reference genomes and human virome, we assembled a non-redundant (at 90% sequence identity) set of 367 RT sequences of a minimum length of 200 AA (among the 155 RT sequences identified from the reference genomes, only three are shorter than 200 AA). A phylogenetic tree of these RT sequences shows that most DGR systems identified in the HMP datasets share similar RTs with the reference genomes; however, there are still some small clades that only contain sequences from the human virome and (or) human microbiome (e.g., the Branch 1 and Branch 2 highlighted in Figure 2), indicating the importance of metagenomic studies in enriching the genomic diversity in human microbiome.

**Figure 3:**
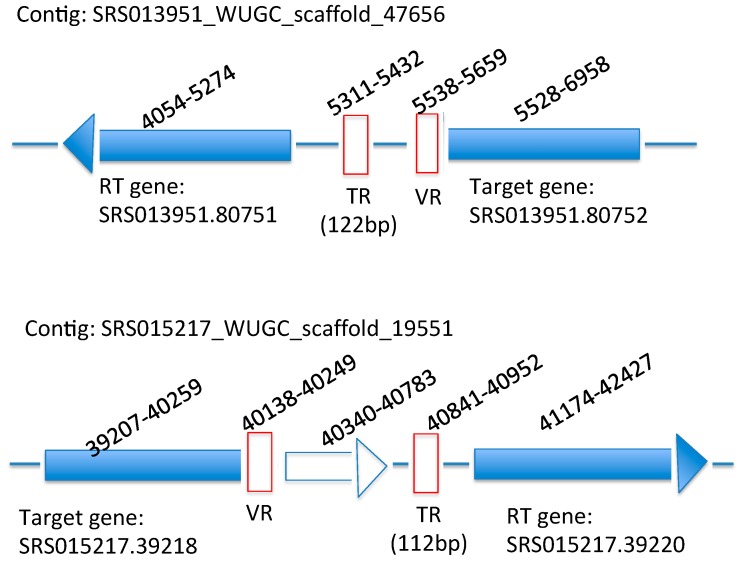
Diagrams of two new DGR systems identified from the human microbiome project (HMP) datasets. Note that the RT genes encoded in these contigs were annotated with unknown functions in the human microbiome project (HMP) annotations.

Figure 3 shows the diagram of two new DGR systems that DGRscan identified from the HMP assemblies. The first one was identified from scaffold SRS013951_WUGC_scaffold_47656 assembled from a stool metagenomic dataset (SRS013951). This scaffold does not share significant sequence similarity with known genomic sequences (in the NCBI NR database) as a whole, although the reverse transcriptase encoded in it (located between 4054 and 5274 bp) shares ~50% sequence identity with the RT in *Firmicutes bacterium* CAG:170. The second DGR system was identified from scaffold SRS015217_WUGC_scaffold_19551 assembled also from a stool metagenomic dataset (SRS015217). Similarly, this scaffold does not share sequence similarity with any of the known genomic sequences as a whole. This DGR system contains an accessory gene between the RT gene and the target gene (but the previous DGR system does not). We note that without applying the proper genomic context information, many of the putative RTase genes we predicted would otherwise remain as hypothetical proteins without annotations or with incorrect or nonspecific annotations. For example, the two putative RTs described above were annotated as proteins of unknown function in the HMP annotations.

### 2.3. Identification and Annotation of the Target Genes

Considering that some target genes may be found in contigs without RT genes and, therefore, may be missed by the *de novo* prediction of the DGR systems, we extracted the TRs (template sequences) from the *de novo* predicted DGR systems and combined them with TRs extracted from the known DGR systems identified from the complete genomes [12] and the human virome dataset [7]. Using these TRs as the reference, we applied DGRscan again to the HMP contigs, to identify complete and partial DGR systems (each must contain a TR–VR pair, in which the TR is similar to one of the reference TRs, but may lack an RT gene, or target gene, or both). From identified DGR systems, we predicted target genes that contain variable regions in the *C*-terminus of their protein products (within a buffer region of 60 bps); the *C*-terminal location of the VR is a common feature shared by DGR variable proteins [6,20].

In total, we identified 1392 genes (and their proteins) that are putative DGR target genes: These genes contain VR sequences at the ends that encode for the *C*-terminus of their protein products and are therefore potentially subject to diversification by the DGR systems. By contrast, only 566 target genes were identified by *de novo* prediction of DGR systems. After removing the redundancy, there are 651 target genes sharing no more than 90% sequence identity at the amino acid level. Not surprisingly, most of these genes—596 in total (92%) encoding for hypothetical proteins, including 565 “hypothetical proteins”, 19 “putative uncharacterized protein (fragment)”, six “conserved hypothetical protein” and six “conserved domain protein”—were shown as encoding for “hypothetical protein” in the HMP annotations. Considering that a similarity search against hidden Markov models of protein domains may provide more sensitive annotations [21], we used hmmscan [22] to annotate our collection of putative DGR target genes. In total, hmmscan provided annotation for 206 proteins (35%), a significant improvement as compared to the HMP consortium annotations (in which only 8% of the genes have some annotations). This fraction (35%) is still less than the fraction of target proteins that have PFAM annotations in the previous study on complete genomes (50%) [5], reflecting that the human microbiome is less studied as compared to the complete genomes.

For the 206 proteins with PFAM annotations, we focus on those to which at least five proteins were assigned (see Table 2). Several PFAM domains, including DUF1566, FGE-sulfatase and DUF3988, are shared between our annotations of the putative variable proteins and the previous study of putative variable proteins identified from complete genomes [5]. However, the frequencies of these domains are different between our study and the previous study: the most common domain is DUF1566 (contained in 47 proteins), followed by FGE-sulfatase (in 42 proteins) in our study; by contrast, 45 proteins are found to contain the FGE-sulfatase domain, but only seven proteins contain the DUF1566 domain in the previous study [5]. We argue that our data reveals the distribution of the different domains involved in the DGR systems in the human microbiomes and, therefore, better reflects the potential importance of the DUF1566 domain to the adaptability of the human microbiome to their hosts. In addition, DUF1566 and the other domain, Fib_succ_major, share similarity according to HHsearch [23]. Both domains are found in the DGR systems in our study, further suggesting that they not only share similar sequences, but also similar structures and biological functions. We also found Big_2 (bacterial Ig-like fold group 2) and Big_3 (bacterial Ig-like fold group 3) in our collection of putative variable proteins, which were not reported in the previous study [5].

**Figure 4:**
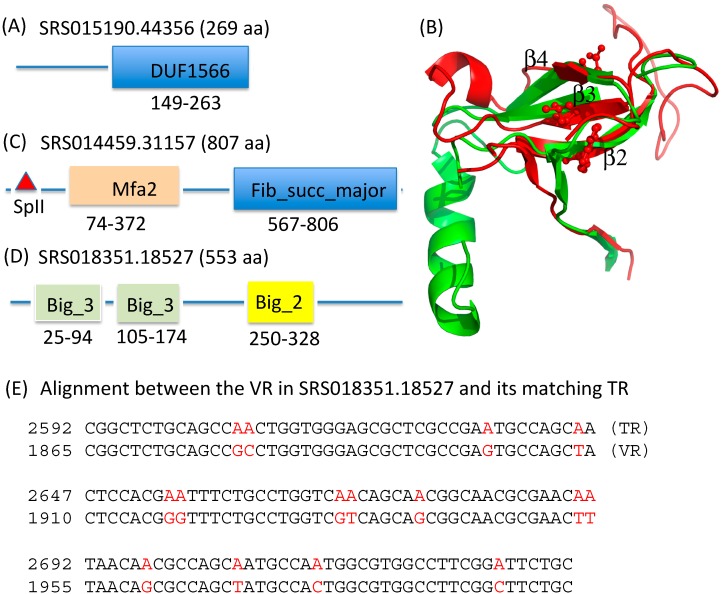
Representative variable proteins identified from the HMP datasets. (**A**) SRS015190.44356 with a DUF1566 domain; (**B**) Superimposition between 2Y3C (TvpA; shown in red) and 4EPS (green); (**C**) SRS014459.31157, a predicted lipoprotein with Mfa2 (shown in bisque) and Fib_succ_major (blue) domains; (**D**) The domain architecture of SRS018351.18527 with bacterial immunoglobin-like domains; and (**E**) the alignment between the VR region in this gene and the matching TR, which contains 15 substitutions (highlighted in red), all involving adenines in the template region.

Here, we show detailed analyses for a few putative variable proteins. The first protein (SRS015190.44356) was predicted from a stool metagenome (SRS015190). The hmmscan prediction reveals a DUF1566 domain near the *C*-terminus of this protein (see Figure 4A). As the VR region is found at the end of the gene, we can conclude that domain DUF1566 in this protein is subject to diversification by the DGR system. The I-TASSER server [24] and RaptorX server [25,26] both predicted that this protein is structurally similar to the structure of a hypothetical protein (BACOVA_04982) from *Bacteroides ovatus* ATCC 8483 (PDB ID: 4EPS), but with very low sequence identity (<20%). Interestingly, 4EPS does not show global similarity with the known variable protein (TvpA) found in *Treponema denticola* (PDB ID: 2Y3C) [6]; however, their *C*-terminus (where the potentially variable residues are located) are strikingly similar (FATCAT [27] reported 44 equivalent positions with an RMSD of 2.97 Å between the *C*-terminus of these two structures of 70 residues), with similar arrangements of β-strands (see Figure 4B). Our analysis suggests that the VRs in these proteins with the DUF1566 domain may share a similar structure with the VR in TvpA (and, therefore, Mtd, as well), despite very low sequence similarity between these VR regions.

The second protein, SRS014459.31157, was also predicted from a stool metagenome (SRS014459). This protein contains a Fib_succ_major domain and an Mfa2 domain (Figure 4C). Mfa2 domains are found in fimbrillin-A-associated anchor proteins Mfa1 and Mfa2, which are typically lipoproteins. Consistent with this, SRS014459.31157 is predicted to be a lipoprotein by the LipoP server [28], which reveals a SpII (signal peptidase II) lipoprotein signal peptide in this protein (see Figure 4C). This protein has 807 amino acids, longer than the typical Mfa1 and Mfa2 proteins (between 300 and 400 residues). Fib_succ_major domain is located at the end of this protein, so it is likely to be the region in this protein that is under diversification of the DGR system.

The third protein SRS018351.18527 contains three bacterial immunoglobulin (Ig)-like domains (two Big_3 domains and one Big_2 domain; see Figure 4D). Only a few instances are known of protein folds that tolerate massive sequence variation for the sake of binding diversity: The immunoglobulin fold is the most extensively characterized instance, and the other instance is the C-type lectin (CLec) fold, as found in the major tropism determinant (Mtd), the target gene of the first discovered DGR system in the *Bordetella* phage. Although we cannot conclude that the immunoglobulin fold is also involved in the diversification by DGR systems (as the predicted Ig-like domains are not located at the end of the protein), it is still interesting to see the connection between the DGR system and the Ig-like fold. We show the alignment between the VR region (the nucleotide sequence) and its matching TR region, which contains 15 substitutions, all involving As in the TR region (Figure 4E), typical for a TR–VR pair.

**Table 2:** Breakdown of the functions (PFAM domains) assigned to the putative variable proteins identified from the HMP datasets.

Pfam Domain	Description	Number of Proteins	Example
DUF1566	Protein of unknown function; similar to Fib_succ_major	47	SRS015190.44356
FGE-sulfatase	Sulfatase-modifying factor enzyme 1	42	SRS052027.8709
Fib_succ_major	*Fibrobacter succinogenes* major domain	9	SRS014459.31157
DUF3988	Found by clustering human gut metagenomic sequences	9	SRS077730.37228
Big_2	Bacterial Ig-like domain (group 2)	9	SRS018351.18527
DUF3751	Phage tail-collar fiber protein	8	SRS013687.79027
CotH	Members of this family include the spore coat protein H	6	SRS016989.185
Big_3	Bacterial Ig-like domain (group 3)	6	SRS018351.18527
CarboxypepD_reg	Carboxypeptidase regulatory-like domain	5	SRS015663.100342

### 2.4. Wide Distribution of the DGR Systems in the Human Microbiome

The 765 DGR systems with RT were identified from 160 HMP assemblies (some dataset contains multiple DGR systems). Therefore, 22% (160 out of 743) of the human metagenomes we checked contained at least a DGR system. Although still a small ratio, it is significantly greater than the ratio of complete genomes that contain DGRs (155 DGR systems were identified from >6000 complete genomes). However, many of the HMP assemblies are rather small (e.g., the smallest assembly contains 4022 bps only), a result of shallow sequencing and imperfect assembling. Among the 160 DGR systems, only one was found in a metagenome of <33 Mbps. When only considering HMP assemblies of at least 33 Mbps, the ratio of samples with DGR systems increased to 37%. Further, among the 10% largest metagenomes, 62% of them each contain at least one DGR system (the ratio is 67% if we also consider partially identified DGR systems that do not contain RT genes), indicating that the DGR systems are rather common in microbial communities, even though they are rare in sequenced bacterial genomes.

## 3. Experimental Section

### 3.1. DGRscan

A minimal DGR system is comprised of a gene encoding reverse transcriptase, a template sequence and a variable region within a target gene. Some DGR systems may target multiple genes. For a complete or draft genome, we may expect to predict a complete DGR system with all of the components (with one or more target genes, each containing a variable region that is a non-perfect repeat of the template region). However, when the input genomic sequences (e.g., metagenome assemblies) are fragmented, we may only be able to predict some components, but not all, of the DGR systems (partial DGR systems) in these sequences.

We devised the DGRscan based on the structural features of DGR systems, to allow both *de novo* identification (based on the prediction of potential template-variable region pairs) and similarity search-based identification of DGR systems using known template sequence as the reference (Figure 1). For the *de novo* prediction mode, DGRscan relies on finding segments in a given genomic sequence that potentially form a TR–VR pair that differs at positions mostly involving As in the TR segment. Specifically, two repeats that are similar to each other spanning at least 60 bp with 7 or more substitutions involving As in one of the repeats (only a small fraction of the substitutions (<30%) are allowed to be involved in non-As in the same repeat) are considered a potential TR–VR pair. The copy with As in the mismatch positions is the template region, and the other copy is the VR. A dynamic programming algorithm was used for the alignment. For speedup, a full dynamic programming is called only when a seed match of at least 60 bps (without indels) is found between two segments. Further speedups are needed for making DGRscan practical for large input genomic sequences (such as complete genomes). One implementation speedup is to search for potential RTase genes in input genomic sequences and then only to look for a potential TR–VR pair in the neighborhood of identified RTase genes (within 10 kbp at both ends). The other speedup strategy is to apply AAY filtering so that only the regions (of 60 bps) containing at least 3 AAYs (Y can be C or T) will be further checked—it has been shown that hypervariation was targeted to 5'-AAY-3' asparagine codons, which allows maximal chemical diversification of the encoded amino acids, while avoiding the formation of stop codons [7,16]. We tested involved parameters empirically and selected the ones that work well for the identification of the DGR systems in complete genomes. Users can choose to turn off the speedup options.

DGRscan can also be guided to search for DGR systems that share similar template sequences with known ones (the similarity search-based mode). This option is useful when the input genomic sequences are fragmented (such as metagenomic sequencing reads or assemblies), as DGRscan can still report partial DGR systems using the existence of the template regions as a clue, even for the cases when no *RTase* genes can be found or no TR–VR pairs can be found (due to the fragmented nature of input sequences).

### 3.2. The Human Virome Datasets and the Human Microbiome Project (HMP) Datasets

We downloaded the whole metagenome assemblies (HASM) and gene annotations (HMGI) for the HMP datasets from the DACC website [29]. In total, there are 743 human metagenome assemblies. The human virome datasets were downloaded from the NCBI website.

### 3.3. Availability of DGRscan and Results

DGRscan was implemented in Python and is available for download from the DGRscan website [30] or Github [31]. The BLAST suite is the only required software by DGRscan; it is used for similarity searches for potential RT and TR regions. DGR systems identified using DGRscan in the HMP datasets are available for download at the DGRscan website.

## 4. Conclusions

Using DGRscan, which was developed by us, we were able to identify many new DGR systems and their associated RT genes in the human microbiomes. We were also able to identify putative target genes (and their protein products) that potentially are under diversification through the DGR systems. Our study shows that DGR systems are of low incidence, but they are ubiquitously found in the human microbiomes and are likely to be important to the adaptability of the human microbiome to the environments in the human hosts. We note that our study only gives a conservative estimation of the distribution of DGR systems in human microbiomes, considering that the shotgun sequencing might not be deep enough to cover the rare species that may carry those systems, and the assemblies of metagenomes are far from perfect.

We have previously shown that genomic context is important for annotating the bacterial immune systems, the CRISPR-Cas system [32]. In this study, we show another appealing application of using the genomic context for the improved annotation of proteins. We were able to annotate many of the hypothetical proteins as either reverse transcriptases or target genes that are under diversification of the DGR systems.

## References

[1] Doulatov S., Hodes A., Dai L., Mandhana N., Liu M., Deora R., Simons R.W., Zimmerly S., Miller J.F. (2004). Tropism switching in *Bordetella* bacteriophage defines a family of diversity-generating retroelements. Nature.

[2] Guo H., Tse L.V., Nieh A.W., Czornyj E., Williams S., Oukil S., Liu V.B., Miller J.F. (2011). Target site recognition by a diversity-generating retroelement. PLoS Genet..

[3] Alayyoubi M., Guo H., Dey S., Golnazarian T., Brooks G.A., Rong A., Miller J.F., Ghosh P. (2013). Structure of the essential diversity-generating retroelement protein bAvd and its functionally important interaction with reverse transcriptase. Structure.

[4] Medhekar B., Miller J.F. (2007). Diversity-generating retroelements. Curr. Opin. Microbiol..

[5] Schillinger T., Zingler N. (2012). The low incidence of diversity-generating retroelements in sequenced genomes. Mob. Genet. Elem..

[6] Le Coq J., Ghosh P. (2011). Conservation of the C-type lectin fold for massive sequence variation in a Treponema diversity-generating retroelement. Proc. Natl. Acad. Sci. USA.

[7] Minot S., Bryson A., Chehoud C., Wu G.D., Lewis J.D., Bushman F.D. (2013). Rapid evolution of the human gut virome. Proc. Natl. Acad. Sci. USA.

[8] Barrangou R., Fremaux C., Deveau H., Richards M., Boyaval P., Moineau S., Romero D.A., Horvath P. (2007). CRISPR provides acquired resistance against viruses in prokaryotes. Science.

[9] Arambula D., Wong W., Medhekar B.A., Guo H., Gingery M., Czornyj E., Liu M., Dey S., Ghosh P., Miller J.F. (2013). Surface display of a massively variable lipoprotein by a Legionella diversity-generating retroelement. Proc. Natl. Acad. Sci. USA.

[10] (2012). The Human Microbiome Project Consortium. Structure, function and diversity of the healthy human microbiome. Nature.

[11] (2012). The Human Microbiome Project Consortium. A framework for human microbiome research. Nature.

[12] Schillinger T., Lisfi M., Chi J., Cullum J., Zingler N. (2012). Analysis of a comprehensive dataset of diversity generating retroelements generated by the program DiGReF. BMC Genomics.

[13] Li W., Godzik A. (2006). Cd-hit: A fast program for clustering and comparing large sets of protein or nucleotide sequences. Bioinformatics.

[14] Howard M.T., Satoshi M. (1970). Viral RNA-dependent DNA polymerase: RNA-dependent DNA polymerase in virions of *Rous sarcoma* virus. Nature.

[15] Pfam RVT_1 Domain. http://pfam.xfam.org/family/PF00078.

[16] Minot S., Grunberg S., Wu G.D., Lewis J.D., Bushman F.D. (2012). Hypervariable loci in the human gut virome. Proc. Natl. Acad. Sci. USA.

[17] Edgar R.C. (2004). MUSCLE: A multiple sequence alignment method with reduced time and space complexity. BMC Bioinform..

[18] Price M.N., Dehal P.S., Arkin A.P. (2009). FastTree: Computing large minimum evolution trees with profiles instead of a distance matrix. Mol. Biol. Evol..

[19] Han M.V., Zmasek C.M. (2009). PhyloXML: XML for evolutionary biology and comparative genomics. BMC Bioinform..

[20] McMahon S.A., Miller J.L., Lawton J.A., Kerkow D.E., Hodes A., Marti-Renom M.A., Doulatov S., Narayanan E., Sali A., Miller J.F. (2005). The C-type lectin fold as an evolutionary solution for massive sequence variation. Nat. Struct. Mol. Biol..

[21] Finn R.D., Clements J., Eddy S.R. (2011). HMMER web server: Interactive sequence similarity searching. Nucleic Acids Res..

[22] The HMMER Server. http://hmmer.janelia.org/search/hmmscan.

[23] Pfam DUF1566. http://pfam.xfam.org/family/Duf1566.

[24] Zhang Y. (2008). I-TASSER server for protein 3D structure prediction. BMC Bioinform..

[25] Kallberg M., Margaryan G., Wang S., Ma J., Xu J. (2014). RaptorX server: A resource for template-based protein structure modeling. Methods Mol. Biol..

[26] The RaptorX Server. http://raptorx.uchicago.edu/.

[27] Ye Y., Godzik A. (2003). Flexible structure alignment by chaining aligned fragment pairs allowing twists. Bioinformatics.

[28] Juncker A.S., Willenbrock H., von Heijne G., Brunak S., Nielsen H., Krogh A. (2003). Prediction of lipoprotein signal peptides in Gram-negative bacteria. Protein Sci..

[29] DACC Website. http://www.hmpdacc.org/.

[30] DGRscan Website. http://omics.informatics.indiana.edu/mg/DGRscan.

[31] DGRscan at Github. https://github.com/YuzhenYe/DGRscan.

[32] Zhang Q., Doak T.G., Ye Y. (2014). Expanding the catalog of cas genes with metagenomes. Nucleic Acids Res..

